# An In Vitro Model of Mast Cell Recruitment and Activation by Breast Cancer Cells Supports Anti-Tumoral Responses

**DOI:** 10.3390/ijms21155293

**Published:** 2020-07-26

**Authors:** Angélica Aponte-López, Jennifer Enciso, Samira Muñoz-Cruz, Ezequiel M. Fuentes-Pananá

**Affiliations:** 1 Unidad de Investigación en Virología y Cáncer, Hospital Infantil de Mexico Federico Gómez, Ciudad de Mexico 06720, Mexico; angibel06@hotmail.com; 2 Programa de Doctorado en Ciencias Biomédicas, Universidad Nacional Autónoma de Mexico (UNAM), Ciudad de Mexico 04510, Mexico; jenny_gr8@msn.com; 3 Centro de Investigación Biomédica de Oriente, Delegación Puebla, Instituto Mexicano del Seguro Social, Delegación Puebla 74360, Mexico; 4 Unidad de Investigación Médica en Enfermedades Infecciosas y Parasitarias, Centro Médico Nacional Siglo XXI, Instituto Mexicano del Seguro Social, Ciudad de Mexico 06720, Mexico

**Keywords:** mast cells, breast cancer, tumor stroma, anti-tumor functions, IFNγ

## Abstract

Breast cancer (BrC) affects millions of women yearly. Mast cells (MCs) are common components of breast tumors with documented agonistic and antagonistic roles in tumor progression. Understanding the participation of MCs in BrC may lead to new therapies to control tumor growth. In this study, we looked into mechanistic models of MC responses triggered by BrC cells (BrCC), assessing both early degranulation and late transcriptional activities. We used aggressive and non-aggressive BrCC to model the progressive staging of the disease over HMC1 and LAD-2 human MC lines. We found that both MC lines were chemoattracted by all BrCC, but their activation was preferentially induced by aggressive lines, finding differences in their active transcriptional programs, both at basal level and after stimulation. Among those genes with altered expression were down-regulated *SPP1*, *PDCD1*, *IL17A* and *TGFB1* and up-regulated *KITLG* and *IFNG*. A low expression of *SPP1* and a high expression of *KITLG* and *IFNG* were associated with increased overall survival of BrC patients from public databases. The set of altered genes is more often associated with tumor stromas enriched with anti-tumoral signals, suggesting that MCs may participate in tumor control.

## 1. Introduction

Breast cancer (BrC) is an important health problem mainly affecting women of productive age. In 2018, the World Health Organization estimated more than two million new cases, representing 11.6% of all cancers and the second place in incidence [1]. Although we have significantly improved BrC diagnosis, classification and treatment, the incidence of the disease has been steadily increasing, and patients often do not respond to traditional chemotherapy. Today, new approaches for cancer treatment include strengthening the immune system to enforce tumor control. Indeed, BrC is generally infiltrated by a variety of immune cells that execute both agonistic and antagonistic roles in tumor progression [2,3], with some immune cells playing well-characterized roles. For instance, macrophages favor extracellular matrix degradation and tumor cell invasion, which is associated with poor clinical outcomes. On the contrary, CD8 T cells favor tumor cell cytotoxicity and their presence is associated with good prognosis [2,3,4]. A clear understanding of the molecular mechanisms displayed by immune cells within the tumor stroma will lead to new tools for cancer treatment.

Mast cells (MCs) are also common components of the breast tumor stroma [2,3,5]. MCs are multifunctional tissue-resident cells of the immune system, that upon activation, and depending on the type of stimuli and receptor involved, release three distinct classes of biologically active compounds: (1) preformed compounds that are stored in cytoplasmic granules, (2) neoformed compounds derived from arachidonic acid oxidation, and (3) neosynthesized compounds derived from transcriptional activation and protein synthesis [6,7]. Preformed biological mediators are released within seconds to minutes, by a process known as degranulation, which involves compound exocytosis and is better understood for anaphylactic responses. Neoformed and neosynthesized compounds are released by different mechanisms of secretion, up to days after MCs activation. The most common preformed bioactive molecules are histamine, heparin, and the proteases tryptase and chymase; while neoformed compounds are mainly prostaglandin and leukotrienes, and neosynthesized compounds include different cytokines, chemokines, and growth factors [8,9]. The selective release of specific granule compounds also occurs in a process sometimes referred to as piecemeal, mainly mediated by vesicular trafficking, indicating discriminatory mechanisms of secretion of the MC cargo [8].

MCs originate in the bone marrow and are released into blood as precursor cells that finish their maturation in tissues [10]. This pathway of MCs ontogeny is dependent on different growth factors and cytokines, of which stem cell factor (SCF) is perhaps the most important [6], a biomolecule often secreted by tumor cells [11]. HMC1 and LAD-2 cells are human lines derived from patients with MC leukemia. Although both cell lines have been extensively used to assess MC biology, they exhibit critical functional differences [12,13,14]. Perhaps the major differences between them are that HMC1 has a constitutively active mutation in the SCF receptor c-Kit (also known as CD117), and therefore is SCF independent; additionally, it does not express functional receptors for IgE. On the other hand, LAD-2 is SCF-dependent, abundantly expresses the high-affinity IgE receptor (FcεRI) and is enriched with preformed granules, and thus it is a good model to examine signals for early degranulation. Less clear are differences in the selectivity of the de novo synthesis of bioactive compounds that are targeted by late mechanisms of release.

The MC role in cancer progression is controversial; while some studies associate high tumor MC density with BrC subtypes of good prognosis or with favorable clinical outcomes [5,15,16,17,18,19,20,21,22], others find them associated with aggressive features [23,24,25,26,27,28,29,30,31]. One potential problem of current approaches to elucidate the participation of MCs in BrC progression, is that most studies are observational and correlative, and until now the molecular mechanisms have not been elucidated. In this study, we first characterized HMC1 and LAD-2 lines using an array of inflammatory mediators to assess potential diverging transcriptional programs between them. We then characterized whether both cell lines were chemoattracted and activated by aggressive and non-aggressive BrC cell (BrCC) lines, and assessed how their transcriptional programs were altered after this activation. We found that both MCs lines were activated by BrCC but observed significant differences in their active transcriptional programs, both at basal level and after stimulation. Interestingly, chemoattraction was induced by all BrCC, but activation was preferentially induced by aggressive BrCC, leading to a significantly altered expression of genes in both MC lines. Among those genes with altered expression were down-regulated *SPP1*, *PDCD1*, *IL17A* and *TGFB1* and up-regulated *KITLG* and *IFNG*. We observed that these transcriptional changes are more often related to anti-tumoral responses, suggesting that MCs can participate in tumor control. Moreover, low expression of *SPP1* and high expression of *KITLG* and *IFNG* were associated with an increased overall survival (OS) in BrC patients from public databases. Elucidation of the MC-selective synthesis and release of bioactive compounds may inform us about MC mechanisms that favor or impede tumor progression. 

## 2. Results

### 2.1. HMC1 and LAD-2 Exhibit Differential Basal Expression Levels of Genes Associated with Cancer and Immunity

**Figure 1:**
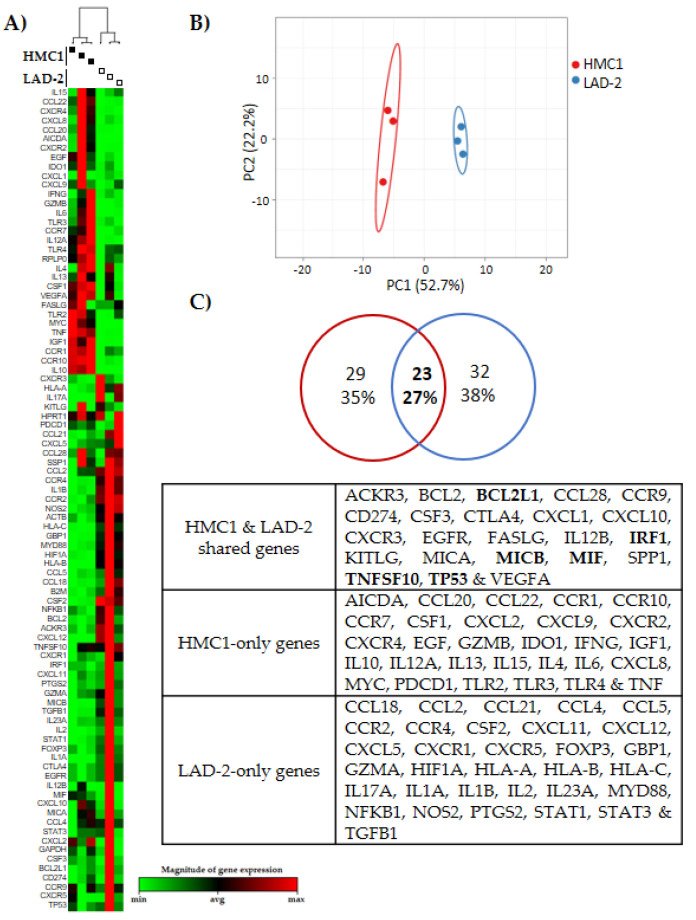
Transcriptional differences between HMC1 and LAD-2 mast cell lines. (**A**) Heat map and dendrogram, and (**B**) principal component analysis comparing the basal expression of 84 genes associated with cancer and immunity. (**C**) Venn diagram showing the number and percentage of genes differentially expressed or shared between both cell lines, and the identity of the genes. Genes in bold are highly expressed genes in both cell lines. Data represent three independent experiments.

To have a better picture of HMC1 and LAD-2 cells similarities/differences at the basal level of transcription of critical genes for inflammation and cancer, we analyzed both MCs using the “Cancer, Inflammation and Immunity Crosstalk” RT-PCR Array. This array measures the expression of 84 genes classified according to their biological functions, mainly as (a) chemokines and chemokine receptors, (b) interleukins/cytokines, (c) growth factors, (d) immunoregulatory or immunosuppressive genes and (e) apoptosis. The array provides five housekeeping genes, and we used the NormFinder Software to determine the most stable reference genes for transcription data normalization (Supplementary Table S1). After gene expression normalization, a non-supervised hierarchical clustergram, heat map and principal component analysis (PCA) showed that both MC lines significantly differ forming separated clusters (Figure 1A,B), only sharing the expression of 27% (23/84) of the genes analyzed, whereas 35% (29/84) were genes basally expressed only in HMC1, and 38% (32/84) were LAD-2-only genes (Figure 1C). Of those shared genes, we observed that *BCL2L1*, *IRF1*, *MICB*, *MIF*, *TNFSF10* and *TP53* were highly expressed in both MCs, having a Ct lower than 23, which is similar to the Ct of the housekeeping genes.

### 2.2. Breast Cancer Cells Induce Mast Cells Chemoattraction and Low-Level Degranulation

Considering the variety of bioactive compounds in their content, MCs have the potential to significantly alter their microenvironment, while being influenced by the array of stimuli enriched in a particular tumor stroma. We used both MCs to experimentally model interactions with BrCC, assuming that we would get different responses from them as suggested by their distinct transcriptional profiles. We used four BrC lines, MCF7 and T47D cells that have an epithelial, terminally differentiated phenotype, are not invasive and do not metastasize in transplanted mice; MDA-MB-231 and Hs578T cells that have a mesenchymal, stem-like phenotype, are invasive and metastasize in mice [32,33]. The former two cell lines were derived from patients with non-aggressive luminal A tumors, while the latter two were derived from aggressive triple negative tumors. Thus, we used these cells lines to model the MC response to BrCC with different aggressive properties and the influence of the progressive staging of the disease. 

**Figure 2:**
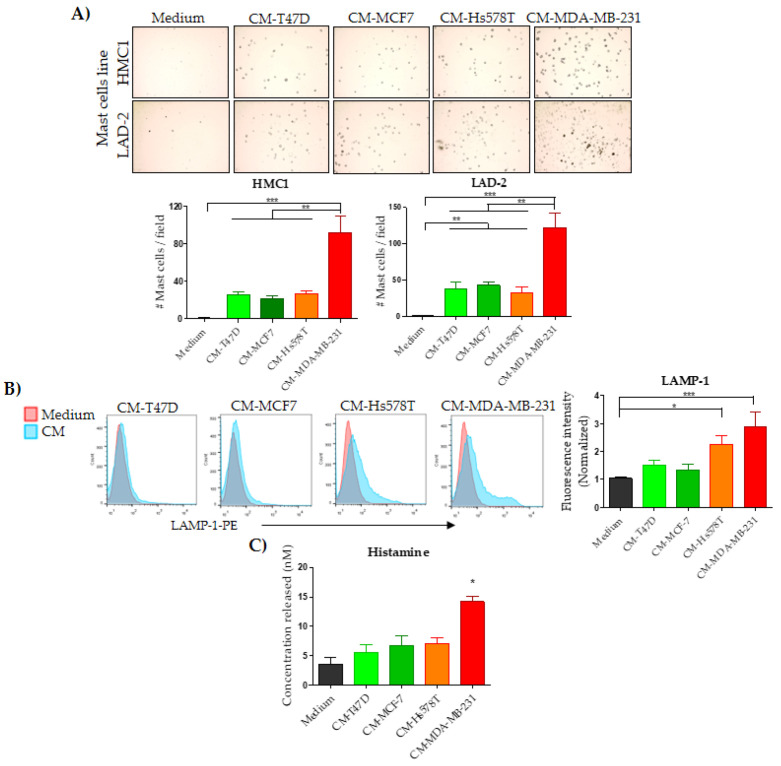
Analysis of chemoattraction and degranulation in response to stimuli derived from breast cancer lines. (**A**) Representative image (top) and their respective plot (bottom) of chemoattracted mast cells in response to the conditioned media (CM) derived from the indicated breast cancer cell lines. (**B**) Representative histogram of LAMP-1 deposition on LAD-2 cells outer membrane (left) and plot (right). (**C**) Histamine release from LAD-2 cells stimulated with the indicated CM. Basal medium was used as negative control. Plotted data represent the mean ± SEM from four independent experiments by duplicate. * *p* < 0.05, ** *p* < 0.01 and *** *p* < 0.001.

We first explored whether conditioned media from BrCC could promote chemoattraction of MCs, explaining the MCs infiltration in the stroma of breast tumors. We performed migration assays using transwell plates, observing that both MC lines were chemoattracted by all the conditioned media, with aggressive MDA-MB-231 cells inducing a significantly higher MC migration than the other BrCCs (Figure 2A). To evaluate whether BrCC could activate MCs and induce their early degranulation, we measured the translocation of the lysosome-associated membrane protein 1 (LAMP-1) to the extracellular membrane of MCs [34] and the histamine release induced by the BrCC-derived conditioned media. Only LAD-2 cells were used for the degranulation analysis since HMC1 are immature cells and thus poorly granulated. In this early activation response, we observed that only the aggressive BrCCs Hs578T and MDA-MB-231 induced significantly higher degranulation than the unstimulated MCs (Figure 2B). In comparison with Hs578T, the MDA-MB-231 induced the largest increment in LAMP-1 translocation. Of note, substance *p* stimulation induced a LAMP-1 translocation almost 10-fold higher than that induced by the aggressive tumor cells (Supplementary Figure S1A), perhaps suggesting that massive degranulation is not a dominant mechanism of MCs activation in the tumor stroma. Rather, a piecemeal mechanism of degranulation with the selective secretion of mediators, without granule-to-plasma membrane fusions, may occur [8,35]. To confirm the mast cell activation, we measured histamine release as a reliable marker for degranulation. The conditioned media from MDA-MB-231 induced the MC secretion of 14.18 nM of histamine, which was significantly higher than that from untreated MCs (3.52 nM) (Figure 2C), whereas the conditioned media from Hs578T triggered a moderate histamine release (7.07 nM), not significantly higher than the basal release. This result is consistent with the higher LAMP-1 translocation induced by MDA-MB-231 cells. The non-aggressive BrCCs T47D and MCF7 induced a non-significant histamine secretion of 5.58 and 6.71 nM, respectively (Figure 2C). Compound 48/80 (1 μg/mL) was used as a positive control and caused the secretion of 43.65 nM of histamine, 3-fold higher than that induced by MDA-MB-231 (Supplementary Figure S1B). Tryptase is one of the main components of pre-formed granules, therefore we also determined the level of tryptase released by MCs upon activation with BrCC-derived conditioned media. We found an average of 35.24 pg/mL induced by non-aggressive cells, similar to basal release (average of 34.93 pg/mL), and lower than aggressive BrCCs (average of 53.43 pg/mL); still, this difference was not significant (Supplementary Figure S1C). Taken together, these data support that breast tumors, and particularly aggressive breast tumors, secrete biomolecules able to attract and activate low level MC degranulation.

### 2.3. Rantes, SDF1, G-CSF, GM-CSF, MCP1, IL8 and SCF Participate in Chemoattraction but Not in Degranulation of Mast Cells

**Figure 3:**
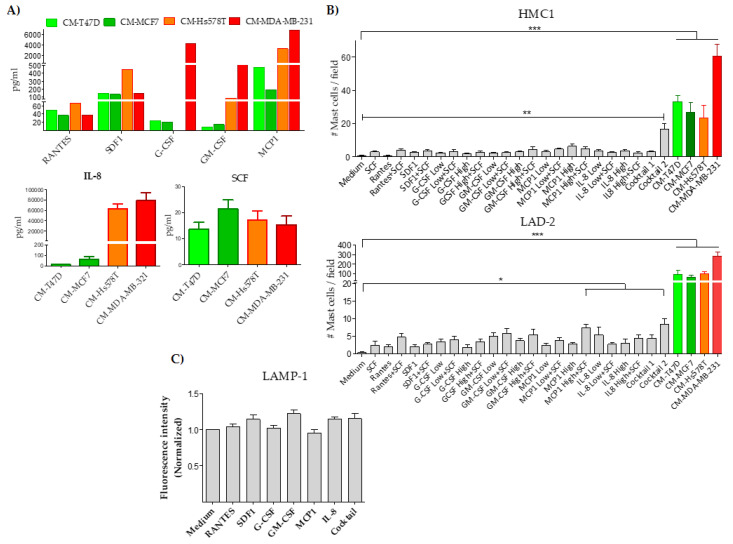
Analysis of mast cell chemoattraction and degranulation by different cytokines. (**A**) The levels of Rantes, SDF1, G-CSF, GM-CSF, MCP1 (top), and IL-8 and SCF (bottom) were measured in the conditioned media (CM) derived from BrCC lines using a multiplex luminex assay (top) and ELISA (bottom). All proteins were at undetectable levels in control media. (**B**) Analysis of chemoattraction of HMC1 (top) and LAD-2 (bottom) cells using recombinant proteins as chemotactic factor and CM as experimental controls. Low and High refers to the mean of the cytokine concentration found in CM derived from non-aggressive cells and aggressive MDA-MB-231 cells, respectively (specified in the methods section). Cocktail 1 and 2 are the combination of all cytokines at low and high concentrations, respectively. (**C**) Analysis of degranulation of LAD-2 mast cells in response to recombinant proteins at high concentrations, individually or combined (cocktail 2). Results are shown as mean fluorescence intensity normalized to unstimulated MCs. Data represent the mean ± SEM from four independent experiments by duplicate. * *p* < 0.05, ** *p* < 0.01 and *** *p* < 0.001.

Different molecules can trigger activation, migration or both, in either MCs or other myeloid cells. Among the most important are RANTES, SDF1, G-CSF, GM-CSF, MCP1, IL-8 and SCF [36,37,38]. We analyzed these proteins in the conditioned media of BrCC to potentially explain mast cell chemoattraction to the tumor stroma. Figure 3A shows that all BrCC secreted similar levels of RANTES, SDF1 and SCF independently of their aggressiveness, whereas the levels of G-CSF, GM-CSF, MCP-1 and IL-8 were markedly higher in the conditioned media from aggressive cells (Figure 3A). To determine if chemoattraction of MCs was induced by these molecules, we performed migration assays using recombinant proteins as chemoattractants. These proteins were used individually, combined with SCF, or in cocktails of all of them. For RANTES, SDF1 and SCF, the concentration used was the mean of the concentration found in the conditioned media of all BrCC, whereas G-CSF, GM-CSF, MCP1 and IL-8 were used at two different concentrations, one corresponding to the levels found in the conditioned media of non-aggressive BrCC (low) and the other to the levels found in aggressive cell conditioned media (high). In general, conditioned media from BrCC showed higher chemoattractant activity than any of the recombinant proteins, alone or in combination. Interestingly, the combination of all biomolecules in cocktail 2 (high concentration) attracted significantly more HMC1 cells compared with the control medium, whereas cocktail 2, as well as the combination of MCP1 at high concentration with SCF, significantly attracted more LAD-2 cells (Figure 3B), indicating that these molecules when are produced by BrCC may drive chemotaxis of MCs into the tumor. Lipid compounds have also been shown to mediate MC chemotaxis [39]. We determined whether the BrCC express the enzymes responsible for the synthesis of lipid mediators within the pathway of prostaglandins, thromboxanes and leukotriens. To this end, we evaluated *PTGS2*, *ALOX12*, *ALOX5AP*, *ALOX5*, *PTGES*, *PTGDS*, *TBXAS1* and *LTC4S* transcriptional data from the Cancer Cell Line Encyclopedia (CCLE) [40]. We only found evidence of expression of *PTGES* and *LTC4S,* but it was not specifically upregulated in MDA-MB-231 cells (Supplementary Figure S2). Thus, these data do not fully explain the mast cell chemotaxis. Next, we tested the capacity of RANTES, SDF1, G-CSF, GM-CSF, MCP-1 and IL-8 to degranulate MCs. Contrary to the chemoattraction effect on MCs, none of the recombinant molecules tested, alone or in combination, induced MCs degranulation (Figure 3C). Thus, although some of these molecules may participate in the MC chemoattraction observed with the conditioned media of aggressive BrCC, other biomolecules are most likely also involved.

### 2.4. MDA-MB-231 Aggressive Cells Activate a Late Mast Cell Transcriptional Program

**Figure 4:**
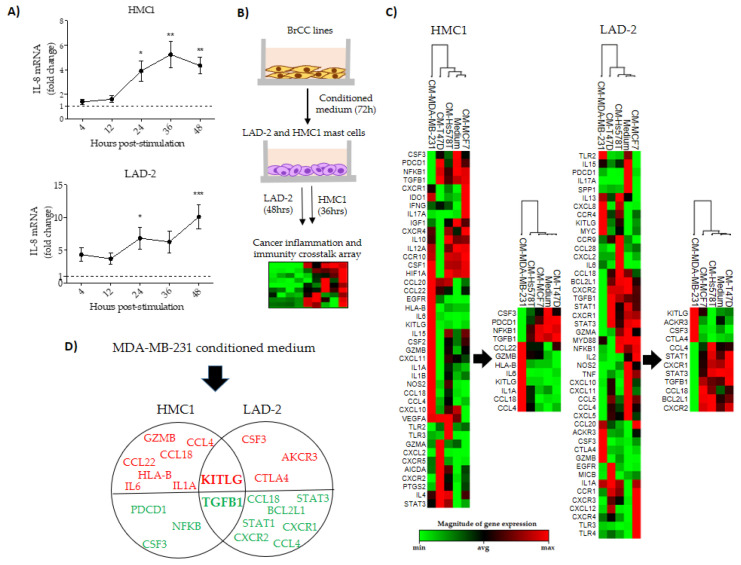
Transcriptional analysis of breast cancer stimulated mast cells. (**A**) Kinetics of *IL-8* expression in HMC1 (top) and LAD-2 (bottom) mast cells in response to the MDA-MB-231 cell-conditioned medium. (**B**) Flow chart of the experimental design used to evaluate the expression array on stimulated mast cells. (**C**) Unsupervised heat map and dendrogram of HMC1 (left) and LAD-2 (right) mast cells in response to conditioned media from the breast cancer cell (BrCC) lines. The simplified heat maps and dendrograms show the principal genes responsible for clustering the response to MDA-MB-231 cells. (**D**) Venn diagram of LAD-2 and HMC1 cells showing uniquely expressed or shared by both mast cell lines after stimulation with MDA-MB-231 cells. Red and green genes represent up-regulation and down-regulation, respectively. In (**A**), data represent the mean ± SEM from three independent experiments by duplicate, while in (**C**,**D**) data represent three independent experiments. * *p* < 0.05, ** *p* < 0.01 and *** *p* < 0.001.

Since BrCC induce MCs chemoattraction, but not a potent early degranulation, we assessed whether they were able to modify the MCs transcriptional profile. We used again the expression array of inflammatory and cancer crosstalk molecules to evaluate the potential of MCs to influence the tumor microenvironment. We first assessed whether we could find evidence of MC transcriptional changes by measuring the kinetic of *IL-8* expression upon MC stimulation with the MDA-MB-231-conditioned media (Figure 4A). We observed that expression peaked at 36 h in HMC1 cells and at 48 h in LAD-2 cells. We also evaluated the kinetics of expression of *VEGF* and *IL-5* finding similar results in *IL-5*, while expression of *VEGF* did not change over time (Supplementary Figure S3). Based in these results we selected 36 h of stimulation for HMC1 cells and 48 h for LAD-2 cells to analyze the expression array (Figure 4B). In agreement with the greater effect observed in chemoattraction and degranulation assays, a non-supervised analysis also showed that stimulation with aggressive MDA-MB-231 cells induced a differential transcriptional program in both MC lines, clustering within themselves and separating them from the stimuli of the other BrCC lines (Figure 4C). A supervised analysis identified 12 genes responsible for MDA-MB-231 clustering, although the genes altered in HMC1 mast cells (*GZMB*, *CCL4*, *CCL22*, *CCL18*, *HLA-B*, *IL-6*, *IL-1A*, *KITLG*, *PDCD1*, *NFKB*, *CSF3* and *TGFB1*) were different than those altered in LAD-2 cells (*CSF3*, *AKCR3*, *CTLA4*, *KITLG*, *CCL18*, *STAT3*, *BCL2L1*, *STAT1*, *CXCR1*, *CXCR2*, *CCL4* and *TGFB1*). Only up-regulation of *KITLG* and down-regulation of *TGFB1* was common in both stimulated MCs (Figure 4D). Because of the diverseness of the altered genes found in the array, analysis of transcription factors, construction of functional interacting networks and pathway enrichment analysis did not show novel information about biological processes relevant to the progression/regression of cancer beyond pathways involved in cytokine signaling and inflammatory responses. Supplementary Figure S4 shows an example of this analysis with MDA-MB-231 cells in which SPI1, STAT3, TP63, CCCTC-binding factor (CTCF) and androgen receptor (AR) appear to be transcription factors altered in MCs.

### 2.5. Mast Cells Altered Genes Mark Increased Overall Survival of Breast Cancer Patients

**Figure 5:**
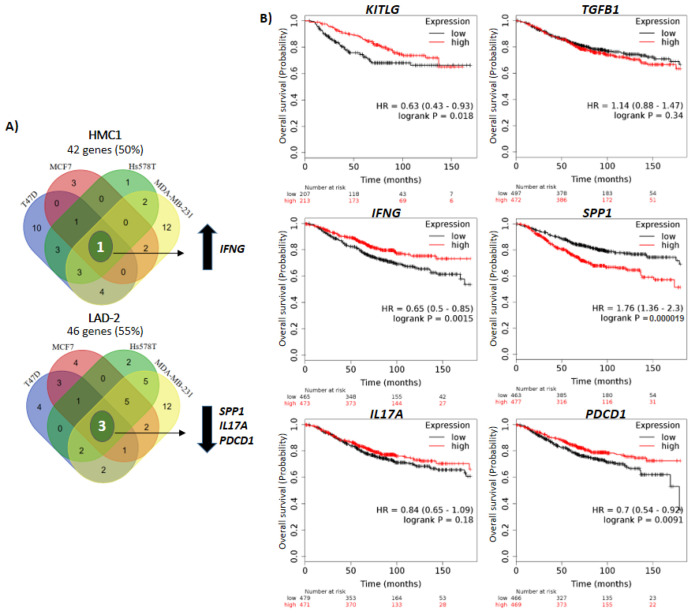
Overall survival analysis of breast cancer patients. (**A**) Venn diagrams of HMC1 (top) and LAD-2 (bottom) showing the intersection of genes changing after stimulation with the breast cancer cell conditioned media. The blue, red, green and yellow zones contain the number of genes changing by conditioned media derived from T47D, MCF7, Hs578T and MDA-MB-231, respectively. Arrows up and down indicate increased and decreased expression, respectively. (**B**) Overall survival plots comparing high or low expression of relevant genes found in the array analysis using breast cancer public databases. In (**A**) data represent three independent experiments.

We observed transcriptional changes in both MCs in about 50% of the genes evaluated after activation with conditioned medium from at least one of the BrCC (Figure 5A). The stimulation with all BrCC shared the up-regulation of one gene (*IFNG*) in HMC1 mast cells, and down-regulation of three genes (*IL17A*, *PDCD1* and *SPP1*) in LAD-2 cells (Figure 5A). Interestingly, although there are not similarities in the genes commonly changing in HMC1 and LAD-2 cells, IFNγ has been associated with anti-tumoral responses and IL17A, PD-1 (encoded by *PDCD1*) and Osteopontin (OPN, encoded by *SPP1*) mostly with the opposite, so are SCF (encoded by *KITLG*) and TGF-β1, respectively (observed in Figure 4D). Using the Kaplan–Meier plotter platform, we analyzed the OS of BrC patients in public databases of the Gene Expression Omnibus (GEO), the European Genome-phenome Archive (EGA), and the Cancer Genome Atlas (TCGA) according to the expression levels of *KITLG*, *TGFB1*, *IFNG*, *IL17A*, *PDCD1* and *SPP1* genes. Indeed, we observed *IFNG* (HR = 0.65, 95% CI = 0.5–0.85, *p* = 0.0015) and *KITLG* (HR = 0.63, 95% CI = 0.43–0.93, *p* = 0.018) as genes of good prognosis, and *SPP1* as of poor prognosis (HR = 1.76, 95% CI = 1.36–2.3, *p* = 0.000019). Remarkably, and contrary to what we expected, *PDCD1* seemed a good prognosis factor when up-regulated, while *TGFB1* and *IL17A* expression levels did not seem to influence the OS of BrC patients (Figure 5B). Altogether, these findings argue that MCs can be attracted and activated by aggressive BrCCs, while the genes changing in activated MCs seem to agree better with a tumor stroma enriched with anti-tumoral signals rather than with signals that facilitate tumor progression.

## 3. Discussion

Mast cell infiltration is commonly found in human BrC and their densities have been associated with either the promotion or suppression of tumor growth [5,17,18,20,22,23,24,25,26,27,29,31,41]. Among the main documented pro-tumoral mechanisms are the promotion of angiogenesis, invasion and metastasis. Less is known about the mechanisms involved in the anti-tumor function of MCs. Through a microscopic analysis of human BrC tissues, one study reported that peritumoral MCs showed cytolytic activity against tumor cells [19]. In addition, the MC-derived proteoglycan heparin inhibited the clonogenic growth of human BrCC when cocultured with fibroblasts [42]. Anti-tumoral functions were also evidenced in MCs activated with IgE antibodies directed against the HER2 antigen, which displayed tumoricidal activity against HER2-positive BrCC [21]. It is possible that particular activities of MCs may reflect particular mechanisms of activation influenced by differences in the stage, grade and subtype of BrC. Additionally, different subtypes of MCs with different intracellular cargos or with specific locoregional location in the tumor, may favor particular anti- or pro-tumoral responses. 

Although solving this great variety of combinations and emerging functions requires a towering effort, here we looked into mechanistic models of MCs response, assessing the capacity of BrCCs to attract them, and trigger early degranulation and late transcription activity. MCs can be recruited by various inflammatory stimuli within the tumor microenvironment, including hypoxia, cellular injury, tissue ischemia and soluble factors secreted by tumor cells and non-cancerous stromal cells. Still, it is not clear what the origin of MCs infiltrating the tumor stroma is, whether they are derived from the local expansion of terminally differentiated resident MCs, or from chemoattraction of neighboring tissue-resident MCs and/or bone marrow-derived mast cell precursors. To the best of our knowledge, this is the first study evidencing that factors secreted by BrCC, preferentially those secreted by aggressive cancer cells, are chemotactic for MCs and trigger an effector response, providing evidence for the latter mechanism.

SCF is the best characterized chemotactic factor for MC recruitment to healthy tissue [7,36]. Similarly, tumor-derived SCF mediates MC infiltration in tumors [11,43]. Although we observed that all BrCC secrete SCF, and it was chemotactic for both LAD-2 and HMC1 cells, particularly in combination with MCP1 or with other chemokines, these data did not explain the chemotactic capacity of BrCC, arguing for the existence of additional chemoattracting factors. CCL15 has been suggested to mediate MC infiltration in colorectal cancer [44], but we were not able to test this cytokine. SCF, SDF1, RANTES and MCP1 have been documented as MCs chemoattractors [36,45], but at concentrations significantly higher than the ones observed in the BrCC-derived conditioned media. Thus, although some of these molecules may participate in the recruitment of MCs, most likely other biomolecules are also involved. 

MC degranulation of preformed biomolecules is a complex regulated process, and different activation signals induce distinct degranulation strategies. For example, IgE-mediated degranulation, a key mechanism of innate defense against helminths, is characterized by the massive release of the granule content. In contrast, the alternative pathway called piecemeal degranulation, triggered mainly by neuropeptides, cytokines and some microbial products, involves the selective release of specific biomolecules [8,35]. This process has been identified during chronic pathological processes, such as Crohn’s disease, angiosarcoma, urticaria and melanoma [8,35,46]. Cancer is a chronic disease, therefore this process may be the preferential mechanism of MC activity in the tumor microenvironment. Here, we observed moderate levels of degranulation and secretion of histamine and tryptase in response to aggressive BrCC, suggestive of a piecemeal mechanism; however, this needs to be confirmed with electron microscopic studies. Similar to our results, a gastric cancer study found that neither G-CSF, GM-CSF, TGF-β, M-CSF, IL-1β, IL17A, IL-6, TNFα, IL10, IFNγ and IL22 were able to induce MC degranulation, while only adrenomedullin did [47]. We do not know if BrCC secrete this component. 

The HMC1 and LAD-2 basal level of transcription has also been explored in different studies. Sven Guhl and collaborators analyzed the expression of seven hallmark genes of MCs [12]: the three chains forming the FcεRI receptor complex (*FCER1A*, *FCER1B* and *FCER1G*), tryptase (*TPSAB1*), chymase (*CMA1*), c-kit (*KIT*) and histidine decarboxylase (*HDC*). The authors found that *TPSAB1*, *FCER1A*, *FCER1G*, *FCER1B* and *CMA1* were either more expressed or exclusively expressed in LAD-2 cells, whereas they did not find HMC1 only genes. Aikaterini Detoraki et al. evaluated the expression of several members of the VEGF family and their receptors [48]: *VEGFA*, *VEGFB, VEGFC*, *VEGFD*, placental growth factor (*PlGF*), *VEGFR1* and *VEGFR2*. This was not a quantitative study, and while most molecules were found in both cell lines, *PIGF* was exclusive of HMC1 cells. In agreement, our data support similar basal levels of *VEGFA* expression in both MC lines. The expression of the nitric oxide synthase (NOS) family has also been compared in both cell lines: nNOS (*NOS1*, neuronal), iNOS (*NOS2*, inducible) and eNOS (*NOS3*, endothelial) [49]. The authors found high expression of *NOS3* and absent expression of *NOS2* in both MCs lines, whereas *NOS1* was only expressed in HMC1 cells. Because of its importance in immune cell responses, we only measured *NOS2* observing low levels of basal expression in both MCs. Gene expression signatures have been recently used to characterize and identify immune cells infiltrating the stroma of various tumors [3,40,50,51,52,53,54]. Although, in general, these studies do not reach a consensus on an MC characteristic signature, they concur in that these cells share expression of *TPSAB1*, *CMA1*, *CTSG*, *CPA3*, *HDC*, *MS4A2* and *PRG2* genes. While only a handful of inflammatory genes were assessed in those studies, some of them are shared with the genes found in our analysis. For instance, we also observe expression of *PTGS1*, *IL1A* and *IL1B*, although they belong to LAD-2 only genes. Perhaps this only reflects that LAD-2 cells appear to be more differentiated cells, similar to the MCs that inhabit the tumor stroma. In summary, these studies agree with our data, further supporting that HMC1 and LAD-2 MCs exhibit significant differences at basal levels of transcription of effector genes.

Our results showed up-regulation of *KITLG* and down-regulation of *TGFB1* in both MCs lines stimulated with MDA-MB-231 aggressive BrCC. *KITLG* high expression identified BrC patients with increased OS. SCF is the most important molecule for MC development, survival and activation. A recent proteomic analysis of plasma samples found lower levels of SCF in women that later developed BrC than in those that remained healthy, supporting SCF as a circulating biomarker, and indirectly suggesting an enhanced tumor controlling mechanism of SCF-activated MCs [55]. On the other hand, TGF-β1 relevance in cancer stems from its association with the epithelial to mesenchymal transition (EMT) [56], an embryonic program in which cells lose expression of adherent proteins and gain expression of movement proteins. Cancer processes arising from EMT-active programs include invasion, metastasis and relapse, and therefore TGF-β is in general considered a bad prognosis marker. We were surprised that we were unable to find an association of TGF-β with the OS of BrC patients. However, multiple lines of evidence place TGF-β also as a tumor suppressor, for instance as an inhibitor of cell proliferation and cell immortalization, and a promoter of apoptosis [57,58]. Remarkably, TGF-β has also been implicated in murine and human gut MC chemoattraction and activation [59,60], altogether perhaps creating a feedback loop that levels TGF-β pro- and anti-tumoral forces.

We also observed HMC1 up-regulation of *IFNG* and LAD-2 down-regulation of *SPP1*, *IL17A* and *PDCD1* induced by all BrCC. While *IFNG* and *PDCD1* were associated with enhanced OS, *SPP1* was associated with the opposite. PD-1 is a checkpoint protein that balances immune responses protecting from uncontrolled immune activation [61]. Cancer cells often induce PD-1 expression to attenuate tumor immunity [62], and the blockade of this checkpoint with antibodies has been extensively used to treat melanoma patients, enhancing CD8 T cell-mediated tumor destruction. However, according to our data, *PDCD1* expression associates with an enhanced OS in BrC patients. One potential explanation for this observation is that, despite PD-1 expression, BrC-infiltrating CD8 T cells retain a robust capacity for the production of effector cytokines IFNγ and TNFα, enhanced proliferation and cytotoxic capacity [63]. In agreement, PD-1 blockade triggers only modest responses in BrC patients, benefiting only a minority of patients [64]. Although PD-1 has not been studied in MCs, *PDCD1* deletion in myeloid cells induces antitumor immunity in a mouse model of cancer [65].

Both *SPP1* and OPN have been identified as markers for progression and the poor survival of BrC patients, promoting tumor cell proliferation, angiogenesis, migration, invasion and bone metastasis [66,67,68]. In agreement, the inhibition of OPN significantly decreased both local tumor growth and distant metastasis in a xenograft murine model [69]. OPN also increased EMT-related transcription factors in BrCC, including Twist, Snail and Slug [70]. A meta-analysis of 10 clinical studies correlated high OPN levels in serum and tumor with the poor survival rates of BrC patients [71]. Additionally, a case-control study found that high *SPP1* expression was associated with increased recurrence of tamoxifen-treated BrC patients [72]. On the other hand, IFNγ is strongly associated with host protection against multiple types of cancers, in experimental models of cancer and in pre-clinical and clinical studies [73,74]. IFNγ activates direct cytostatic effects on cancer cells [75], as well as triggers a cytotoxic response from CD8 T cells and NK cells, perhaps the most important immune anti-tumor activity [73]. A study in which the IFNγR pathway was restored using CRISPR technology, increased the efficacy of immunotherapy because of increased CD8 T cell function and better antigen presentation [76]. Moreover, IFNγ levels in serum were associated with a favorable clinical response in BrC patients before and after combined anti-CTLA-4 and anti-PD-1 immunotherapy [77]. Taken together, our findings support that MCs can be attracted and activated by BrCCs, particularly by the most aggressive subtypes. Upon activation, we observed altered transcription of *KITLG*, *TGFB1*, *IFNG*, *SPP1*, *IL17A* and *PDCD1*, whose associated functions and expression pattern marked and improved OS of BrC patients, supporting that MCs contribute with a tumor stroma with anti-tumoral functions in BrC. 

## 4. Materials and Methods

### 4.1. Cell Culture and Generation of Conditioned Media

T47D, MCF7, Hs578T and MDA-MB-231 human BrCC lines were obtained from the American Type Culture Collection (ATCC, Manassas, VA, USA). The MCs line HMC1 was kindly donated by the Mayo Clinic, Rochester, MN, USA [13], whereas the LAD-2 line was obtained from the National Institute of Allergy and Infectious Diseases [14]. All cell lines are regularly tested for short tandem repeat profiles to verify their authenticity. T47D and HMC1 cells were cultured in RPMI-1640 medium, MCF7 and Hs578T in Dulbecco’s modified Eagles’s medium (DMEM), MDA-MB-231 in Dulbecco´s modified Eagles´s medium with nutrient mixture F-12 (DMEM/F12), and LAD-2 in STEM-PRO. All cell culture media were purchased from Life Technologies (Grand Island, NY). RPMI, DMEM and DMEM/F12 culture media were supplemented with 10% heat-inactivated fetal bovine serum (FBS), and antibiotic/antimycotic (100 U/mL penicillin, 100 μg/mL streptomycin, 0.25 μg/mL fungizone (Carlsbad, CA, USA). STEM-PRO medium was supplemented with 100 ng/mL of recombinant human SCF (Preprotech, Rocky Hill, NJ), antibiotic/antimycotic and nutrient complement following the manufacturer´s instructions. All cell cultures were maintained at 37 °C in humidified air and 5% CO_2_.

To obtain conditioned media, all BrCC lines were cultured in 182-cm^2^ flasks in their respective media until they reached 80–90% of confluency. Supernatants were discarded and the cell monolayer was washed with phosphate-buffered saline (PBS, Life Technologies, Grand Island, NY, USA) 1X. Subsequently, 25 mL of STEM-PRO supplemented with 1% of nutrient complement (low-nutrient medium) was added. Because LAD-2 cells are highly delicate and only proliferate in STEM-PRO medium, and because we wanted to have a uniform stimulus for both MCs lines, all conditioned media were done in STEM-PRO. Conditioned media were collected after 72 h of incubation, centrifuged at 2500 rpm/5 min to eliminate floating cells and used immediately to stimulate the MCs lines. Conditioned media were used diluted 3:1 with fresh media.

### 4.2. Analysis of Cytokines in Conditioned Media

Different cytokines and growth factors known to either chemoattract or activate MCs were measured in the conditioned media of the BrC lines. Levels of Regulated on Activation, Normal T Cell Expressed and Secreted (RANTES/CCL5), stromal cell-derived factor 1 (SDF1/CXCL12), granulocyte-colony-stimulating factor (G-CSF), granulocyte macrophage-colony-stimulating factor (GM-CSF) and monocyte chemoattractant protein-1 (MCP1/CCL2) were measured using the MILLIPLEX HCYTOMAG-60K kit (EMD Millipore, Darmstadt, Germany) [32]. Levels of IL-8 (BD, San Diego, CA, USA) and SCF (Biolegend, San Diego, CA, USA) were determined through Enzyme-Linked ImmunoSorbent Assays (ELISA) and following the manufacturer´s recommended procedure.

### 4.3. Migration Assay

One hundred thousand MCs per experimental condition were resuspended in 200 μL of low nutrient media and placed in the upper chamber of a transwell insert with 6.5 mm diameter and 5-μm pore size (Corning, Kennebunk ME, USA). Transwells were placed in 24-well culture dishes containing 800 μL of conditioned media or low-nutrient basal medium supplemented with RANTES, SDF1, G-CSF, GM-CSF, MCP1, IL-8, and SCF recombinant proteins as chemoattractant factors, either individually or in particular combinations. All these recombinant proteins were obtained from PeproTech, Rocky Hill, NJ, USA. The concentrations of SCF, RANTES and SDF1 used were the mean found in all conditioned media (20 pg/mL SCF, 50 pg/mL RANTES, 200 pg/mL SDF1); G-CSF, GM-CSF, MCP1 and IL-8 were used in two different concentrations: the mean found in non-aggressive cells (defined as low concentration), and the mean found in MDA-MB-231 aggressive cells (defined as high concentration). Low concentrations were: 50 pg/mL G-CSF, 50 pg/mL GM-CSF, 500 pg/mL MCP1 and 50 pg/mL IL-8; while high concentrations were: 5 ng/mL G-CSF, 500 pg/mL GM-CSF, 5 ng/mL MCP1 and 80 ng/mL IL-8. Cocktail 1 and 2 are the combination of all cytokines, as follows. Cocktail 1 composition: SCF (20 pg/mL) + RANTES (50 pg/mL) + SDF1 (200 pg/mL) + G-CSF (50 pg/mL) + GM-CSF (50 pg/mL) + MCP1 (500 pg/mL) + IL-8 (50 pg/mL). Cocktail 2 composition: SCF (20 pg/mL) + RANTES (50 pg/mL) + SDF1 (200 pg/mL) + G-CSF (5 ng/mL) + GM-CSF (500 pg/mL) + MCP1 (5 ng/mL) + IL-8 (80 ng/mL). In each experiment low-nutrient basal medium was used as negative control, whereas culture medium supplemented with 5% of FBS was used as positive control. After 24 h of incubation at 37 °C in a humidified 5% CO_2_ environment, migratory cells were observed in the bottom wells using a digital camera Motic 5.0 MP and the Motic image plus 3.0 software. A total of 4 fields/well in 10× magnification were counted. Three independent triplicates were performed. 

### 4.4. Mast Cells Stimulation

Before stimulation, MCs were allowed to rest in a density of 5 × 10^5^/mL in low-nutrient basal medium for 8 h. After this, the culture medium was discarded and replaced by the conditioned media originating from T47D, MCF7, Hs578T and MDA-MB-231 BrCC, or with fresh low-nutrient basal medium supplemented with RANTES, SDF1, G-CSF, GM-CSF, MCP1, IL-8 and SCF recombinant proteins. Cell cultures were incubated at 37 °C in a humidified 5% CO_2_ environment. To measure degranulation and tryptase release, LAD-2 cells were incubated for 30 min with conditioned media from BrCC. MCs and supernatants were separated by centrifugation at 1500 rpm/5 min; then the mast cells were analyzed by flow cytometry and their supernatants were used to measure the tryptase and histamine released as described below. For each experiment, low-nutrient basal medium was used as negative control and Substance *p* (5 µM; Sigma-Aldrich, Saint Louis, MO, USA) as positive control. For gene expression analysis, mast cells were incubated for 4, 12, 24, 36 and 48 h, and processed as described below.

### 4.5. Flow Cytometry

One hundred fifty thousand MCs were stimulated as previously described, recovered from cultures and maintained at 4 °C during staining. Briefly, to block unspecific binding, cells were incubated with 10% FBS and 3 µL of FcR Blocking Reagent (Miltenyi Biotec, Auburn, CA, USA) for 20 min at 4 °C, then cells were washed with PBS 1X and incubated with phycoerythrin (PE)-conjugated anti-LAMP-1 (Thermo Fisher, San Diego, CA, USA) for 20 min at 4 °C at an 1:100 dilution. After two washes, cells were resuspended in 150 μL of PBS 1× and 3 µL of 7 amino-actinomycin (7AAD, BD Biosciences, Piscataway, NJ, USA) was added. At least 30,000 events/sample were acquired in a Guava flow cytometer (Luminex, Austin Texas, USA). Data analyses were performed using Flowjo_V10 software compensating with single color stains. Doublets and death cells were excluded from analysis using FSC-Height versus FSC-Width and positive 7AAD cells, respectively. Data were normalized to unstimulated mast cells and expressed as fluorescence mean intensity.

### 4.6. Measurement of Histamine and Tryptase Release from Mast Cells

Histamine and tryptase were used as markers of mast cell degranulation and measured in the supernatants of LAD-2 cells stimulated as described above. The levels of histamine and tryptase were quantified by competitive enzyme immunoassay (Cayman Chemicals, Ann Arbor, Michigan, USA) and ELISA (Genway Biotech, San Diego, CA, USA), respectively, according to the manufacturer´s recommended procedure. The amount of both proteins was calculated based on standard curves.

### 4.7. RNA Extraction and Quantitative Real Time PCR

RNA was extracted from 3 × 10^6^ MCs stimulated with the different conditioned media using the RNAeasy Plus kit (Qiagen, Spoorstraat KJ Venlo, Netherlands) and following the manufacturer´s protocol; then, RNA purity and concentration were estimated in a nanodrop One/OneC (Thermo Fisher). Reverse transcription (RT) was performed on 1.2 µg of purified RNA through Reverse first strand cDNA synthesis using RT2 First Strand Kit (Qiagen, Spoorstraat KJ Venlo, Netherlands) according to the manufacturer protocol. *VEGFA*, *IL-5* and *IL-8* expression was measured first to set the time in which mast cells are altering gene expression in response to the BrC stimuli. A qPCR reaction was performed using RT^2^ SYBR Green Mastermix (Qiagen, Spoorstraat KJ Venlo, The Netherlands) in a Rotor-gene Q thermocycler (Qiagen) in the following conditions: initial denaturation at 95 °C for 10 min, followed by 40 cycles of 95 °C for 15 s, 60 °C for 30 s, and 72 °C for 30 s. The amplification reaction was ended at 72 °C for 10 min for a final extension step. The following primers pairs were used: *IL-8* (forward 5′-AGGTGCAGTTTTGCCAAGGA-3′ and reverse 5′-TTTCTGTGTTGGCGCAGTGT-3′), *VEGF* (forward 5′-CTCGATTGGATGGCAGTAGCT-3′ and reverse 5′-GCACCCATGGCAGAAGG-3′), *IL-5* (forward 5′-CGTTTCAGAGCCATGAGGATGC-3′ and reverse 5′-GCCAAGGTCTCTTTCACCATGC-3′), and *GAPDH* (forward 5′-CTTCACCACCATGGAGAAGGC-3′ and reverse 5′-GGCATGGACTGTGGTCATGAG-3′). The cycle threshold (Ct) values were determined by the software supplied with the thermocycler and expression was calculated relative to *GAPDH* using the 2^−ΔΔCt^ method.

Expression of 84 genes related to immune cell responses was evaluated in HMC1 and LAD-2 mast cells using the “Human Cancer Inflammation and Immunity Crosstalk” RT^2^ Profiler PCR Array (PAHS-181Z, Qiagen, Spoorstraat KJ Venlo, Netherlands). The basal and stimulated transcriptional profile of MCs was analyzed. Three independent biological replicates were done for each MC stimulated with conditioned media derived the BrCC. Analysis was carried out after 36 and 48 h post-stimulation for HMC1 and LAD-2 cells, respectively, according to the previous time setting. cDNA was synthesized as described above, then arrays were performed following the manufacturer’s instructions.

### 4.8. Analysis of Gene Expression Signatures

NormFinder V20 software was used to identify the optimal normalization genes among the set of housekeeping genes included in the array. Two genes with the most stable measures were chosen, and then gene expression was calculated relative to those housekeeping genes. To compare the basal levels of gene expression in both MCs lines, we first used HMC1 cells as the control condition and LAD-2 as the experimental condition. Then, we identified genes shared by both MCs lines as those genes with fold change values of less than 2; genes with higher than 2 change values were assigned to LAD-2-only. To identify HMC1-only expressed genes LAD-2 cells were set as control and HMC1 cells as experimental. In stimulated MCs, genes with fold change values higher than 2 over unstimulated cells were considered as significantly altered. To view the altered genes shared by both stimulated MCs, Venn diagrams were made using the Bioinformatics & Evolutionary genomics portal. A non-supervised hierarchical clustergram and heat map of genes with changes in their expression in at least one condition of stimulation were constructed for each MCs line. Data analysis was performed in the web portal “Gene Globe Data Analysis Center” from Qiagen. To visualize the principal component analysis of MCs, we used the web tool Clustvis (https://biit.cs.ut.ee/clustvis/). The mRNA expression levels of *PTGS2*, *ALOX12*, *ALOX5AP*, *ALOX5*, *PTGES*, *PTGDS*, *TBXAS1* and *LTC4S* transcriptional in T47D, MCF7, Hs578T, and MDA-MB-231 were obtained from the Cancer Cell Line Encyclopedia (CCLE) database, which includes detailed genetic information from 1457 human cancer cell lines [40].

### 4.9. Bioinformatic Analyses of Affected Pathways and Processes

To identify MC signaling pathways and biological processes relevant to cancer that were affected upon stimulation with BrCC, we carried out transcription factor enrichment (TFE) analysis, network expansion and an extended pathway enrichment analysis (PEA) with the down and upregulated genes in the LAD-2 and HMC-1 cells after stimulation with the BrCCs (Supplementary Figure S4). Genes with altered expression in both MC lines were grouped for this analysis aiming to uncover any common pathway upstream of the down- or up-regulated genes. TFE and PEA were performed using the online tool Enrichr developed by Ma’ayan’s Lab at the Icahn School of Medicine at Mount Sinai [78,79]. Enrichr uses a list of Entrez gene symbols as input and returns different types of enrichment information retrieved from different databases. For the TFE, we used the ENCODE and ChEA consensus results, and for the PEA we used the KEGG 2019 Human information. Interaction Networks were inferred using the X2Kweb tool. X2Kweb performs a transcription factor enrichment analysis from the input genes, and executes a protein–protein interaction network expansion [80,81]. Using the molecules integrating the interaction networks we performed the extended PEA in Enrichr.

### 4.10. Overall Survival of Breast Cancer Patients

OS plots were performed through the platform “Kaplan-Meier plotter” using data from BrC patients. This platform use gene expression data and OS information from GEO, EGA and TCGA databases [82]. We analyze altered genes identified in MCs: *IFNG*, *SPP1*, *IL17A*, *PDCD1*, *KITLG*, and *TGB1*. For each gene, we choose only JetSet best probe and split genes expression by trichotomization (lower tercile vs. upper tercile) followed a 180-month survival time. The analysis included the following quality controls: Removal of redundant samples and exclusion of biased arrays.

### 4.11. Statistical Analysis

Statistical analysis was performed using the GraphPad Prism 5 software. We first analyzed the data distribution using D’Agostino test. For data in which more than two groups had a normal distribution, one-way analysis of variance (ANOVA) and Tukey test as post-hoc were performed. Data lacking normality and/or homogeneity of variance were analyzed with non-parametric Kruskal–Wallis and Dunnett test as post-hoc assessment. Description of experimental replicates are described in figures legends. The results are shown as mean ± the standard error of the mean (SEM). For the OS analysis, proportional hazard ratios with 95% confidence intervals for a Cox regression model were used. *p* values ≥ 0.05 were considered statistically significant.

## Supplementary Materials

The following are available online at https://www.mdpi.com/1422-0067/21/15/5293/s1. Table S1. Analysis of the housekeeping genes with the most stable expression through all experimental conditions. Figure S1. Comparison of MC degranulation induced by conditioned media (CM)-derived from BrCC and by the MC-secretagogues substance P or compound 48/80 (C48/80). Figure S2. Comparison of *PTGS2*, *ALOX12*, *ALOX5AP*, *ALOX5*, *PTGES*, *PTGDS*, *TBXAS1* and *LTC4S* expression in the breast cancer cell lines. Figure S3. Kinetics of IL-5 (top) and VEGF (bottom) expression in HMC1 and LAD-2 mast cells in response to the MDA-MB-231 cell conditioned medium. Figure S4. Bioinformatic analysis of differential regulated genes in MC lines after stimulation with MDA-MB-231 conditioned medium.

## Abbreviations

BrC	Breast cancer
BrCC	Breast cancer cell
EGA	European Genome-phenome Archive
EMT	Epithelial-mesenchymal transition
G-CSF	Granulocyte-colony-stimulating factor
GEO	Gene Expression Omnibus
GM-CSF	Granulocyte macrophage-colony-stimulating factor
FcεRI	High-affinity IgE receptor
LAMP-1	Lysosome-associated membrane protein 1
MCs	Mast cells
MCP1	Monocyte chemoattractant protein-1
MDSCs	Myeloid-derived suppressor cell
OPN	Osteopontin
OS	Overall survival
PCA	Principal Component Analysis
SCF	Stem cell factor
SDF1	Stromal cell-derived factor 1
TCGA	The Cancer Genome Atlas
TLA	Three letter acronym
