# Supplementary material for: An In Vitro Model of Mast Cell Recruitment and Activation by Breast Cancer Cells Supports Anti-Tumoral Responses
Source: Int J Mol Sci. 2020 Jul 26;21(15):5293. doi: 10.3390/ijms21155293 (PMC7432939; doi:10.3390/ijms21155293)
Supplement: Supplementary file 1 [file ijms-21-05293-s001.pdf]

Table 1. Analysis of the housekeeping genes with the most stable expression through all experimental conditions.

?	Stimulation	Ct				
		**ACTB**	**BM2**	**GAPDH**	**HPRT1**	**RPLP0**
**HMC1**	Medium (1)	11.66	16.49	14.25	19.86	13.07
	Medium(2)	11.75	15.17	14.23	20.3	13.13
	Medium (3)	11.61	16.02	14.28	20.25	13.59
	CM-T47D (1)	11.87	15.58	14.25	20.11	13.17
	CM-T47D (2)	11.6	15.25	13.89	19.97	12.99
	CM-T47D (3)	11.52	15.68	14.43	19.69	13.2
	CM-MCF7 (1)	12.02	15.76	14.28	20.36	13.42
	CM-MCF7 (2)	11.83	15.48	14.24	20.41	13.29
	CM-MCF7 (3)	11.43	15.77	14.2	19.91	13.16
	CM-Hs578T (1)	11.83	15.58	14.36	20.2	13.31
	CM-Hs578T (2)	11.77	15.51	14.42	20.15	13.25
	CM-Hs578T (3)	11.19	15.68	13.91	19.19	12.98
	CM-MDA-MB-231 (1)	11.81	15.66	14.33	20.39	13.54
	CM-MDA-MB-231 (2)	11.62	15.48	14.02	20.42	13.4
	CM-MDA-MB-231 (3)	11.35	15.44	14.36	19.98	13.09
Stability values HMC1		0.046	0.13	0.057	0.107	0.034
		GAPDH + RPLP0 = 0.030				
LAD-2	Medium (1)	11.33	13.94	14.6	20.26	14.44
	Medium(2)	11.66	13.06	14.86	21.5	14.72
	Medium (3)	11.44	13.13	14.76	20.31	14.25
	CM-T47D (1)	11.33	14.1	14.37	20.91	14.83
	CM-T47D (2)	11.77	13.55	14.78	22.04	15.49
	CM-T47D (3)	11.13	14.79	14.68	19.89	14.46
	CM-MCF7 (1)	11.67	14.48	14.76	21.38	15.17
	CM-MCF7 (2)	11.8	13.03	14.64	21.64	14.96
	CM-MCF7 (3)	11.5	14.03	14.85	20.53	15.03
	CM-Hs578T (1)	11.54	14.11	14.66	21.05	14.9
	CM-Hs578T (2)	11.75	13.47	14.62	21.39	14.93
	CM-Hs578T (3)	11.47	13.91	15.3	21.19	15.33
	CM-MDA-MB-231 (1)	11.36	13.39	14.23	19.95	14.14
	CM-MDA-MB-231 (2)	11.88	13.56	14.81	22.15	15.28
	CM-MDA-MB-231 (3)	11.35	13.95	14.96	20.89	14.98
Stability values LAD-2		0.064	0.273	0.057	0.267	0.035
		ACTB + RPLP0 = 0.032				
Stability values HMC1 & LAD-2		0.061	0.35	0.049	0.043	0.24
		GAPDH + HPRT1 = 0.040				

NormFinder V20 software was used to identify the optimal normalization genes among the set of housekeeping genes included in the array. Based on this analysis, the housekeeping genes used to normalize the genes expression in MCs were the following: GAPDH + HPRT1 for both MCs lines (for the combined analysis of figure 1), GAPDH + RPLP0 for HMC1 (for the individual analysis of figures 4 and 5), ACTB + RPLP0 for LAD-2 (for the individual analysis of figures 4 and 5). Ct: cycle threshold, ACTB: actin beta, B2M: beta-2-Microglobulin, GAPDH: glyceraldehyde-3-phosphate dehydrogenase, HPRT1: hypoxanthine phosphoribosyltransferase 1, RPLP0: ribosomal protein lateral stalk subunit P0. The numbers (1), (2), and (3) refer to the three independent experiments.

Commented [M1]:

Commented [M2]:

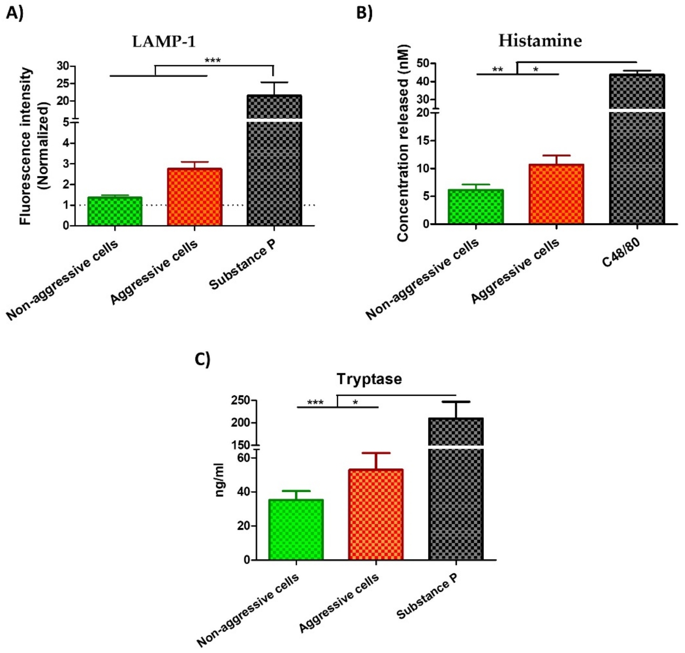

Figure S1. Comparison of MC degranulation induced by conditioned media (CM)-derived from BrCC and by the MC-secretagogues substance P or compound 48/80 (C48/80). (**A**) Detection of LAMP-1 cell surface translocation, (**B**) histamine release and (**C**) tryptase release. Data represent the mean ± SEM from four independent experiments by duplicate. *P<0.05 and ***P<0.001.

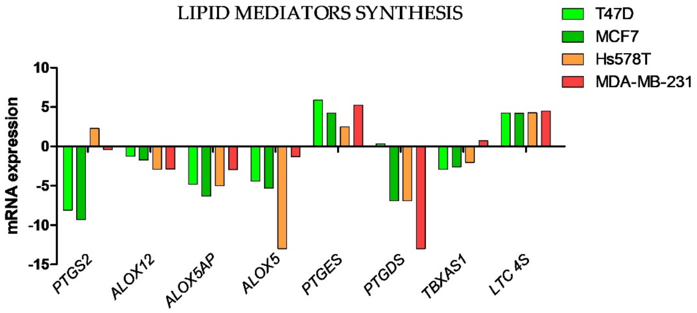

Figure S2. Comparison of *PTGS2, ALOX12, ALOX5AP, ALOX5, PTGES, PTGDS, TBXAS1* and *LTC4S* expression in the breast cancer cell lines. Data were obtained from the Cancer Cell Line Encyclopedia (CCLE) [40].

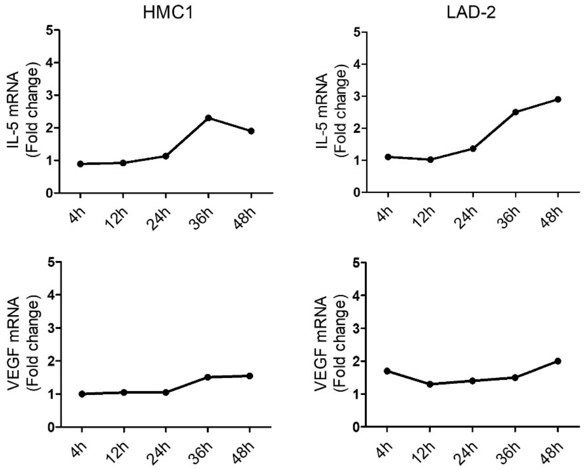

Figure S3. Kinetics of IL-5 (top) and VEGF (bottom) expression in HMC1 and LAD-2 mast cells in response to the MDA-MB-231 cell conditioned medium. Data represent an experiment representative.

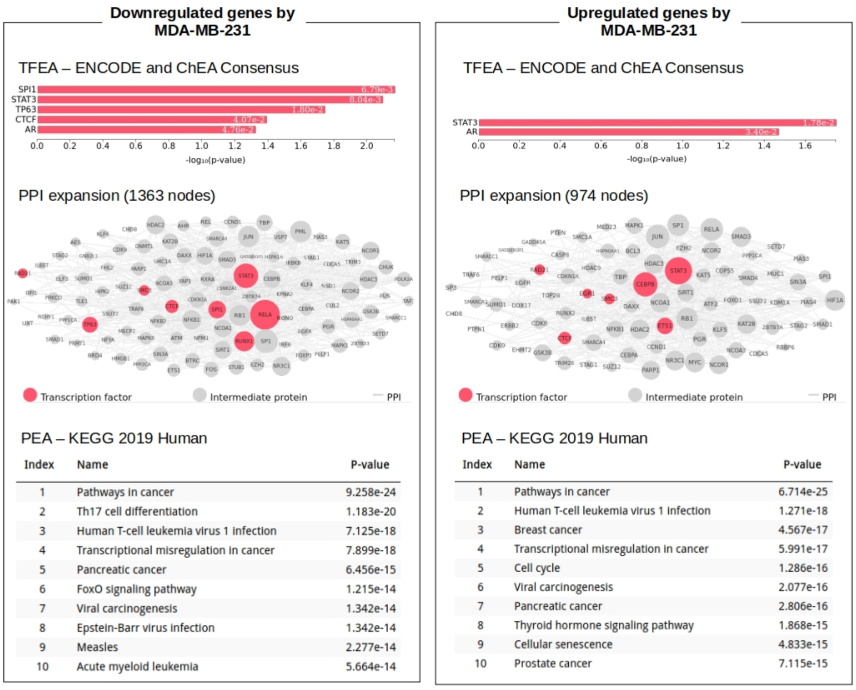

Figure S4. Bioinformatic analysis of differential regulated genes in MC lines after stimulation with MDA-MB-231 conditioned medium. Transcription factor enrichment analysis, protein-protein interaction network expansion and pathway enrichment analysis. The pathway enrichment analysis was performed with the nodes (transcription factors and intermediate proteins) of the interaction networks. Tables resume the top-ten enriched pathways with their corresponding p-value.
